# Parenteral protein-based rotavirus vaccine

**DOI:** 10.1016/S1473-3099(17)30244-X

**Published:** 2017-05-05

**Authors:** Miguel O’Ryan, Benjamin A Lopman

**Affiliations:** Millennium Institute on Immunology and Immunotherapy, Faculty of Medicine, University of Chile, Santiago 8380453, Chile; Department of Epidemiology, Rollins School of Public Health, Emory University, Atlanta, GA, USA

Vaccination is the best method for the prevention of the severe diarrhoeal disease and estimated 215 000 deaths that occur annually due to rotavirus infection.^1^ The first rotavirus vaccine, Rotashield, reached the US market in 1998 but was withdrawn after less than a year following concerns about its association with intussusception.^2^ It took nearly another decade to develop two second-generation vaccines, Rotarix and RotaTeq, both of which are highly efficacious and have a lower risk of intussusception than their predecessor.^3,4^

The Rotarix and RotaTeq vaccines are currently used in national immunisation programmes of over 80 countries and subnationally or in the private sector of many others. Their use has led to impressive reductions in incidence of severe rotavirus diarrhoea by more than 80% in high-income and 50% in low-income settings.^5^ Increasingly, evidence shows reductions in diarrhoea-associated mortality of 31% in infants younger than 1 year and 42% in children younger than 5 years in countries with low child mortality.^6^ Other vaccines have been or will soon be developed, including the Lanzhou Lamb vaccine (China), Rotavin-MI (Vietnam), Rotavac (India), UK bovine strain-based reassortant vaccine (USA, India, and Brazil), and neonatal strain RV3BB (Australia). Clinical trials of these products in India, Ghana, and Niger suggest similar efficacy to Rotarix and RotaTeq in low-income settings.^7–9^ All these vaccines are live human-attenuated or animal-human reassortants administered orally. Like other live oral vaccines such as oral polio, cholera, and typhoid, they are less immunogenic and efficacious in children in low-income settings, probably because of a combination of factors that underpins the infant’s immune response, including maternal antibodies, chronic enteropathy, the microbiota, and interference from other infections. Additionally, a low-level risk for intussusception (in the range of one to seven cases per 100 000 vaccinated infants) has been observed for Rotarix and RotaTeq;^10^ this finding might be due to a class effect of replicating rotavirus vaccines.

In this context, Michelle Groome and colleagues^11^ report the first phase 1/2 study of a novel parenteral rotavirus vaccine for use in infants. The vaccine includes a truncated VP8 subunit protein of the human Wa strain (VP7 serotype 1 and VP4 serotype 8) and a tetanus toxoid P2 protein. Infants were randomly assigned to receive 10, 30, or 60 μg of vaccine with aluminium hydroxide or a saline placebo, coadministered with routine vaccines at ages 6, 10, and 14 weeks. Frequency and severity of adverse events were similar between groups. Adjusted and unadjusted IgG seroresponses against VP8 strains were 98–100%; unadjusted IgA seroresponses were in the range of 58–81% against the P8 protein, but only 9–27% when whole lysate was used. Adjusted neutralising antibody responses were over 80% for P8 strains, 30–50% for P4 strains and 17–23% for P6 strains.

Using similar methodology to that used to assess polio vaccines,^12^ infants received the human attenuated Rotarix vaccine after the last parenteral vaccine dose, and vaccine virus excretion at day 5, 7, and 9 after the first dose was measured by stool ELISA.^11^ Encouragingly, vaccine shedding (any positive sample) was 57% (95% CI 23–76%) lower in vaccinated children (30 μg and 60 μg dose groups combined) than in the children who received placebo. Taking these results together, the authors conclude that the vaccine is immunogenic, and that reduced Rotarix vaccine virus shedding suggests intestinal immunity, which might be a proxy for vaccine efficacy. The authors also acknowledge the absence of significant heterotypic immunity, indicating that studies with vaccines with different P serotypes are needed.

The study is the first phase 2 human trial of an inactivated rotavirus vaccine, and shows the potential of such a strategy, as well as the challenges it faces. First, a non-replicating vaccine approach could possibly circumvent the lower efficacy of live oral vaccines observed in less developed regions, although that remains a conjecture until a study with clinical endpoints is done. Second, a more predictable advantage is that a parenteral vaccine is not expected to cause intussusception. Third, an inactivated vaccine could have some effect on reducing rotavirus shedding but, as for polio, it might be better at preventing disease while less effective at preventing shedding than live oral vaccines.

Groome and colleagues^11^ indicate that a phase 2 trial with a formulation with additional rotavirus antigens is planned, with the hopes of broadening what appears to be a predominantly homotypic response. Hopefully when the phase 3 trial is designed, this vaccine will be compared with an established vaccine in a non-inferiority trial, rather than to placebo. Such a study will be more difficult and will require substantially more infant participants, but would provide more definitive answers regarding comparative efficacy, and might be the only ethical choice with widespread vaccine use. Finally, implementation of a new parenteral vaccine in a crowded vaccine calendar might require antigen combinations, with old and possibly new targets such as norovirus, which is another major cause of childhood diarrhoeal disease.^13^

## References

[1] Tate JE, Burton AH, Boschi-Pinto C, Parashar UD. Global, regional, and national estimates of rotavirus mortality in children <5 years of age, 2000–2013. Clin Infect Dis 2016; 62 (suppl 2): S96–105. 27059362 10.1093/cid/civ1013 PMC11979873

[2] Centers for Disease Control and Prevention. Withdrawal of rotavirus vaccine recommendation. MMWR Morb Mortal Wkly Rep 1999; 48: 1007. 10577495

[3] Ruiz-Palacios GM, Pérez-Schael I, Velázquez FR, Safety and efficacy of an attenuated vaccine against severe rotavirus gastroenteritis. N Engl J Med 2006; 354: 11–22. 16394298 10.1056/NEJMoa052434

[4] Vesikari T, Matson DO, Dennehy P, Safety and efficacy of a pentavalent human-bovine (WC3) reassortant rotavirus vaccine. N Engl J Med 2006; 354: 23–33. 16394299 10.1056/NEJMoa052664

[5] Yen C, Tate JE, Hyde TB, Rotavirus vaccines: current status and future considerations. Hum Vaccin Immunother 2014; 10: 1436–48. 24755452 10.4161/hv.28857 PMC4185955

[6] Burnett E, Jonesteller CL, Tate JE, Yen C, Parashar U. Global impact of rotavirus vaccination on childhood hospitalizations and mortality from diarrhea. J Infect Dis 2017; published online April 18 DOI:10.1093/infdis/jix186.. PMC5543929 28430997

[7] Bhandari N, Rongsen-Chandola T, Bavdekar A, , for the India Rotavirus Vaccine Group. Efficacy of a monovalent human-bovine (116E) rotavirus vaccine in Indian infants: a randomised, double-blind, placebo-controlled trial. Lancet 2014; 383: 2136–43. 24629994 10.1016/S0140-6736(13)62630-6 PMC4532697

[8] Armah GE, Kapikian AZ, Vesikari T, Efficacy, immunogenicity, and safety of two doses of a tetravalent rotavirus vaccine RRV-TV in Ghana with the first dose administered during the neonatal period. J Infect Dis 2013; 208: 423–31. 23599316 10.1093/infdis/jit174 PMC3699001

[9] Isanaka S, Guindo O, Langendorf C, Efficacy of a low-cost, heat-stable oral rotavirus vaccine in Niger. N Engl J Med 2017; 376: 1121–30. 28328346 10.1056/NEJMoa1609462

[10] Aliabadi N, Tate JE, Parashar UD. Potential safety issues and other factors that may affect the introduction and uptake of rotavirus vaccines. Clin Microbiol Infect 2016; 22 (suppl 5): S128–35. 27129416 10.1016/j.cmi.2016.03.007 PMC5839333

[11] Groome MJ, Koen A, Fix A, Safety and immunogenicity of a parenteral P2-VP8-P[8] subunit rotavirus vaccine in toddlers and infants in South Africa: a randomised, double-blind, placebo-controlled trial. Lancet Infect Dis 2017; published online may 5 10.1016/S1473-3099(17)30242-6. PMC7771518 28483414

[12] O’Ryan M, Bandyopadhyay AS, Villena R, Inactivated poliovirus vaccine given alone or in a sequential schedule with bivalent oral poliovirus vaccine in Chilean infants: a randomised, controlled, open-label, phase 4, non-inferiority study. Lancet Infect Dis 2015; 15: 1273–82. 26318714 10.1016/S1473-3099(15)00219-4

[13] Lopman BA, Steele D, Kirkwood CD, Parashar UD. The vast and varied global burden of norovirus: prospects for prevention and control. PLoS Med 2016; 13: e1001999. 27115709 10.1371/journal.pmed.1001999 PMC4846155

